# Comparison of Goal Achievement When Transitioning from In-Person Therapy to Teletherapy in Westchester County Early Intervention Program Due to the COVID-19 Pandemic

**DOI:** 10.5195/ijt.2022.6450

**Published:** 2022-06-03

**Authors:** Inna C. De Leon, JennaLynn Philipps, Marina Yoegel, Joseph Byrnes, Jordan S. Kase

**Affiliations:** 1 School of Medicine, New York Medical College, Valhalla, New York, USA; 2 Children With Special Needs, Westchester County Department of Health, New Rochelle, New York, USA; 3 The Regional Neonatal Center, Maria Fareri Children's Hospital at Westchester Medical Center, Valhalla, New York, USA

**Keywords:** COVID-19, Early intervention, Telehealth

## Abstract

The sudden transition to virtual therapeutic services during the COVID-19 pandemic provided a unique opportunity to explore telehealth as a platform for delivering early intervention (EI) services. Through retrospective chart review of 93 children, we collected the following data: demographics, diagnosed conditions, therapy type, service format, and provider-reported participant goal achievement (1=no progress, 2=little progress, 3=moderate progress, 4=great deal of progress, 5=outcome achieved) over a six-month period before and after transitioning to telehealth. Pre- and post-transition progress scores were compared using the Wilcoxon signed-rank test. Results demonstrated maintained progress among children who transitioned from in-person to virtual services for similar therapy types. Children receiving speech therapy in-person and virtually demonstrated increased achievement (3.00 vs 3.33; p=0.032). Participants receiving a particular therapy post-transition but not in-person attained similar achievement as those who received the same therapy only in-person. Our research suggests that teletherapy may be a viable option for delivering EI services.

The COVID-19 pandemic forced many healthcare organizations to rapidly transition from in-person to virtual services. On March 18, 2020, the New York State (NYS) Early Intervention Program (EIP) implemented telehealth to continue the delivery of necessary therapeutic and support services during the pandemic (New York State Department of Health [NYSDOH], 2020). Accordingly, the Westchester County EIP halted all in-person services and quickly moved to virtual services. Most families and providers in the Westchester County EIP had no experience or training with teletherapy prior to this change. The sudden adoption of teletherapy in response to a public crisis generated a larger pool of EIP participants receiving virtual services. This immediate transition to virtual therapeutic services during the COVID-19 pandemic provided a unique opportunity to explore the effectiveness of telehealth as a platform to deliver early intervention (EI) services.

EI is a group of therapeutic and support services provided to families and children aged birth to three years with developmental delays and disabilities (Centers for Disease Control and Prevention [CDC], 2019). Significant cognitive, language, motor, physical, and socio-emotional growth occurs during the first three years of a child's life (Smythe et al., 2021). EI has been found to influence a child's developmental trajectory and improve outcomes for families and communities (Chiu et al., 2020; Smythe et al., 2021). A delay or interruption in such services may place vulnerable children at risk for further developmental decline or regression (Fung & Ricci, 2020; Murphy et al., 2021).

Telehealth became the ideal solution for continuing essential services while simultaneously prioritizing public safety during the COVID-19 pandemic (Little & Stoffel, 2021). The term “telehealth” refers to a health service delivery model that utilizes information and telecommunication technology with the goal of improving access to care (American Telemedicine Association [ATA], 2020). Telehealth is not a new concept in EI and has been used to address the needs of children with disabilities even before the pandemic (Behl et al., 2017; Cason, 2009; Ingersoll et al., 2016). Several studies have shown high satisfaction among caregivers and providers with regard to the use of telehealth in EI (Blaiser et al., 2013; Cason, 2009; Lalios, 2012; Murphy et al., 2021; Olsen et al., 2012; Wallisch et al., 2019). Commonly cited benefits of telehealth include increased flexibility, improved service coordination, enhanced family engagement through caregiver coaching practices, and greater provider accessibility in remote and rural areas (Behl et al., 2017; Blaiser et al., 2013; Cole et al., 2019; Lalios, 2012; Olsen et al., 2012). Currently, there are only a few studies demonstrating the objective efficacy of teletherapy for children aged 0-3 years (Behl et al., 2017; Blaiser et al., 2013; Constantinescu et al., 2014; Kronberg et al., 2021).

The objective of this study was to explore the longitudinal progress made among Westchester County EIP participants with a heterogeneous group of diagnoses and treatment types who transitioned services from in-person therapy to teletherapy during the COVID-19 pandemic.

## METHODS

A research team from New York Medical College and Maria Fareri Children's Hospital partnered with the Westchester County EIP to implement the study. EIP representatives contacted and obtained written consent from families who met the following criteria: (1) had been enrolled in EI since at least October 2019, (2) had experienced in-person EI services, and (3) had opted to continue EI services via teletherapy in March 2020. A total of 235 families met the previous criteria and were contacted regarding the study. Of the 235 families contacted, 103 families consented to the study, and 132 families declined. 10 consented participants were excluded from the study due to missing information in their EI chart. This is a retrospective cohort study of 93 consented participants.

Via chart review, demographic information, diagnosed conditions for which the child was receiving therapy, therapy type, service delivery format, and participant progress scores were recorded and analyzed using SPSS 27.0 (REF: IBM Corp. Released 2020. IBM SPSS Statistics for Macintosh, Version 27.0. Armonk, NY: IBM Corp). Participant progress was measured using the Likert scale defined by Westchester County EIP (1=no progress, 2=little progress, 3=moderate progress, 4=great deal of progress, 5=outcome achieved). Each goal listed in the child's individualized family service plan (IFSP) for each therapy type received a progress score determined by the child's provider. During enrollment in EI, IFSP progress is mandated to be recorded every 6 months. The score reflects participant progress during the previous six-month specific IFSP period. Only the most recent progress scores before and after the transition period were collected for this study. The transition date was March 18, 2020. Scores reported prior to this transition date were reflective of the 6-month period before the COVID-19 pandemic. Scores reported after March 18, 2020 were reflective of at least 6 months of teletherapy services. An average progress score was calculated if the participant had more than one IFSP goal within a specific therapy discipline. Although some children received other therapy types, only the progress scores for physical therapy (PT), occupational therapy (OT), speech therapy (ST), and special instruction (SI) were collected since these were the most common therapeutic services received by our research participants. SI includes early intervention services focused on helping infants and children achieve major cognitive, adaptive, and socio-emotional milestones. The study participants were categorized into two groups: children who received both in-person therapy and teletherapy for the same therapy types (i.e., received PT in-person and virtually), and children who experienced both service delivery models (in-person and telehealth) transitioning to differing therapy types (i.e., received PT only in-person and received speech therapy only virtually).

### DATA ANALYSIS

The Wilcoxon signed-rank test was used to compare pre- and post-transition period progress scores only among subjects who received both in-person therapy and teletherapy within the same therapy type. The Mann-Whitney U test was used to compare in-person therapy and teletherapy progress scores among subjects who received both in-person therapy and teletherapy but for differing therapy types. Paired t-test was used to compare other continuous variables.

## RESULTS

### PARTICIPANTS

The average age at the inception of teletherapy after the March 18, 2020 transition date was 23 months (SD ± 8). Most participants had been receiving EI services for 8 (SD ± 8) months before transitioning to teletherapy. Sixty-four percent of participants were male; 75.3% of participants were Caucasian; and 72% of families had private or non-Medicaid health insurance. (Table 1) The most common diagnosed conditions were dyspraxia, mixed receptive-expressive language disorder, and delayed milestone in childhood. (Table 2) Some participants had more than one diagnosed condition. All participants received at least one of the following therapeutic services either during in-person therapy and/or teletherapy: PT, OT, ST, and/or SI. (Table 3)

**Table 1:** EI Participant Demographic Information

Age in Months on 3/18/2020 (mean ± SD)		23 ± 8
Time between Program Enrollment and 3/18/2020 in Months (mean ± SD)		−8 ± 8
Gender n (%)	Male	58 (64%)
	Female	32 (36%)
Race/Ethnicity n (%)	White or Caucasian	70 (75.3%)
	Black or African American	5 (5.4%)
	Asian	3 (3.2%)
	Hispanic or Latino	12 (12.9%)
	Unavailable	3 (3.2%)
Insurance Type n (%)	Non-Medicaid	67 (72%)
	Any Medicaid and Non-Medicaid	14 (16.2%)
	Declined to Answer	7 (7.5%)
	Unavailable	4 (4.3%)

**Table 2:** EI Participant Clinical Information

Diagnosed Condition	Number of Participants (%)
Dyspraxia	43 (46.2%)
Mixed Receptive-Expressive Language Disorder	36 (38.7%)
Delayed Milestone in Childhood	25 (26.9%)
Generalized Muscle Weakness	20 (21.5%)
Feeding Difficulties	10 (10.8%)
Autistic Disorder	10 (10.8%)
Unspecified Lack of Coordination	9 (9.7%)
Hearing Loss	3 (3.2%)
Torticollis	3 (3.2%)
Extremely Low Birthweight	2 (2.2%)
Down Syndrome	1 (1.1%)
Angelman Syndrome	1 (1.1%)
Obstructive Hydrocephalus	1 (1.1%)

**Table 3:** Pre- and Post-Transition Period EI Services Participation (Total Subjects n=93)

Therapy Type (n)	In-Person Therapy Only n (%)	Teletherapy Only n (%)	In-Person Transition to Teletherapy n (%)
**Physical Therapy (46)**	14 (30.4%)	7 (15.3%)	25 (54.3%)
**Occupational Therapy (37)**	13 (35.2%)	9 (24.3%)	15 (40.5%)
**Speech Therapy (59)**	15 (25.4%)	23 (39.0%)	21 (35.6%)
**Special Instruction (32)**	10 (31.2%)	10 (31.2%)	12 (37.6%)

### EI SERVICES PARTICIPATION

As seen in the fourth column of Table 3, 35-54% of participants depending on therapy type (PT, OT, ST, or SI) continued to receive the same service type when transitioning from in-person therapy to teletherapy. A small percentage of participants ceased participation in the therapy type they were receiving in-person and initiated a new therapy type after transitioning to teletherapy. When assessing only children who switched service types when transitioning from in-person to virtual treatment, we observed shifts in the percentage of children who received specific types of services. PT and OT reduced the number of children receiving these specific therapies when transitioning from in-person to virtual, whereas ST increased the number of children who were receiving services via telehealth. Of EI participants who received physical therapy at some point (n=46), 30.4% received in-person therapy only, while 15.3% participated exclusively in virtual treatment. For all children who received occupational therapy (n=37), 35.2% utilized in-person services only, whereas 24.3% engaged in solely virtual therapy. Among speech therapy participants (n=59), there were more children who received only virtual services (39%) than only in-person (25.4%). Participation in special instruction remained similar (31.2%) regardless of service delivery format among children who received either only in-person or only virtual treatment. (Table 3)

### GOAL ACHIEVEMENT

Children who continued to participate within the same therapy discipline across both treatment platforms (in-person and teletherapy) maintained their progress and achieved similar scores pre- and post-pandemic for physical therapy (3.67 vs 4.00; P=0.676), occupational therapy (3.00 vs 3.00; P=0.889), and special instruction (3.00 vs 3.00; P=0.326). However, children who continued to receive speech therapy via teletherapy after receiving speech therapy in-person prior to the pandemic showed increased achievement over the observational period in their treatment goals (3.00 vs 3.33; P=0.032). See Table 4.

**Table 4:** Comparison of Progress Scores for Participants Receiving the Same Therapy Type Both In-Person Therapy and Via Teletherapy

Therapy Type	In-Person Therapy Progress Score Median (Range)	Teletherapy Progress Score Median (Range)	P-value	In-Person Therapy Total Goals Median (Range)	Teletherapy Total Goals Median (Range)	P-value
**Physical Therapy (PT)**	3.67 (2.00–5.00)	4.00 (1.67–5.00)	0.676	2 (2–4)	2 (1–5)	0.574
**Occupational Therapy (OT)**	3.00 (2.00–4.00)	3.00 (1.00–4.00)	0.889	2 (2–3)	2 (2–3)	0.914
**Speech Therapy (ST)**	3.00 (1.00–4.67)	3.33 (2.00–4.67)	0.032	3 (2–5)	2 (1–5)	0.049
**Special Instruction (SI)**	3.00 (1.33–4.33)	3.00 (2.00–4.33)	0.326	2 (2–4)	3 (3–5)	0.220

Among children who were receiving some form of therapy via EI in-person and received a different therapy type after they transitioned to teletherapy, similar progress was able to be attained virtually as those who were receiving the particular therapy type in-person as was demonstrated by similar achievement scores whether in-person or virtually. See Table 5.

**Table 5:** Comparison of Progress Scores for Participants Receiving Differing Therapy Types In-Person versus Teletherapy

Therapy Type	In-Person Therapy Progress Score Median (Range)	Teletherapy Progress Score Median (Range)	P-value	In-Person Therapy Total Goals Median (Range)	Teletherapy Total Goals Median (Range)	P-value
**Physical Therapy (PT)**	3.00 (2.00–5.00)	3.00 (1.60–4.60)	0.751	3 (1–4)	3 (1–6)	0.853
**Occupational Therapy (OT)**	3.00 (2.00–4.00)	2.50 (2.00–4.33)	0.435	3 (2–3)	3 (2–6)	0.857
**Speech Therapy (ST)**	3.00 (2.00–4.67)	3.00 (1.33–5.00)	0.359	3 (2–3)	3 (1–5)	0.899
**Special Instruction (SI)**	2.45 (1.00–3.67)	2.59 (1.00–4.00)	0.849	3 (1–5)	3 (3–6)	0.257

### NUMBER OF GOALS

The maximum number of therapy goals among participants who received virtual services was consistently higher than the maximum number of therapy goals among participants who received in-person services for all therapy disciplines. The only exception was among subjects who received speech therapy through both service delivery models (Table 4) where teletherapy experience resulted in fewer therapy goals than in-person experience (P=0.049).

## DISCUSSION

Our research project is one of the first studies to investigate objectively measured EI outcomes via telehealth at the height of the COVID-19 pandemic. We demonstrated that despite the lack of prior training among both families and providers, goal achievement remained similar in transitioning from in-person therapy to teletherapy. In fact, among those children who were receiving the same services across both formats, we demonstrated that those who were receiving speech therapy in person and again virtually continued to progress as evidenced by improvement of their achievement scores during this longitudinal period. We further exhibited that service format did not affect the quality of therapeutic sessions. EI participants who were receiving a new type of therapeutic service virtually were able to achieve similar progress scores as those who were receiving the same therapy type in person.

In comparison to a number of satisfaction-based studies, there is currently a scarcity of objectively measured outcomes-based research on the use of telehealth in EI. In a previous study conducted during the COVID-19 pandemic, Kronberg et al. (2021) found improved child performance in addition to increased caregiver satisfaction and family goal achievement with telehealth coaching. This study provided novel contributions to telehealth research by taking place during a public crisis when sudden adoption of telehealth was necessary, involving community-based EI providers, and including participants with various clinical diagnoses. In contrast to our study, EI providers in this study had the additional benefit of having received training in both telehealth and the caregiver coaching model prior to implementing treatment. However, the results of this study must be considered cautiously due to its smaller sample size of only 17 families. One study investigating the use of telehealth among children who are deaf or hard of hearing, Behl et al. (2017) demonstrated that children receiving telehealth had at least similar outcomes as children receiving in-person therapy. The study was limited by a clinically homogenous sample population and involving participants who only received either in-person therapy or teletherapy not both. Previous studies by Blaiser et al. (2013) and Constantinescu et al. (2014) also concluded that telehealth may lead to similar or better EI participant outcomes; however, both studies are limited by their small, clinically homogenous sample population. Due to the lack of and limitations in currently available outcomes-based studies, more research is needed to better understand the efficacy of telehealth among EI participants.

An important finding of our study is the identification of types of therapy which may be efficaciously delivered both via in-person and telehealth. We found that children who were receiving in-person speech therapy and continued their sessions via telehealth achieved continued progress as evidenced by longitudinally increasing achievement scores over time. This suggests that, for speech therapy in particular, telehealth may be a very appropriate format to provide services. Previous studies support this finding. Behl et al. (2010) found that children who are deaf or hard of hearing had similar or improved language outcomes with consistent telehealth with well-trained speech-language pathologists (SLP). In our study, speech therapy providers had no previous training with telehealth from Westchester County EIP. Not only were those who transitioned from in-person to virtual speech therapy able to continue to progress according to their IFSP results, but those who only received speech therapy via telehealth were able to achieve goals similarly as those who received speech therapy only during the in-person therapy period. A systematic review involving primary school-age children also found that SLP interventions delivered via telehealth resulted in similar or improved progress, though more so with speech sound intervention than with language intervention (Wales et al., 2017). A more recent study that measured speech and lingual skills among patients with a stutter observed positive outcomes with virtual speech therapy sessions (Eslami Jahromi & Ahmadian, 2021). SLP practice mainly focuses on auditory and visual exchange between client and provider and utilizes activities that can be easily converted into real-time videoconferencing (Theodoros, 2012). This may explain why speech therapy may be equally or more achievable with telehealth than with in-person therapy. Benefits expressed by practitioners providing speech therapy virtually among our research group included the ability to zoom up close to their mouths to clearly demonstrate proper lip and mouth movement. Further, caregivers reported the added benefit of recording therapy sessions and replaying them at times when the child was not receiving therapy (Philipps et al., 2021). It is important to consider the possibility that providers and families were seeking out trainings and materials independently of what was provided within EI, which could have resulted in the success of certain therapy types via telehealth, a factor unknown in the current analyses. Additionally, EI participants may have seen different providers with varying levels of telehealth experience during the pandemic, which could have influenced goal achievement.

When considering the number of IFSP goals for each therapy type, in each format, we found that other than for those receiving ST, children receiving telehealth consistently had more therapeutic goals. Only those children receiving ST both in-person and virtually who were able to achieve progressive improvement had fewer IFSP goals virtually than in-person. This continued progress may be a product of not just the format type being appropriate for this discipline of therapy, but also suggests an association between intensity of therapy services and child outcomes. Previous studies have highlighted that time spent practicing an activity correlates with individual performance in that specific activity (Camden, 2020; Palisano & Murr, 2009). Having several goals within a given therapy discipline, especially with time constraints associated with EI services, may result in less time to practice an activity and, thus, reduce the opportunity for progress on a specific goal. Therefore, limiting the number of IFSP goals may help improve EI participant outcomes in a telehealth format. When assessing the number of IFSP goals, it is important to consider the effect of the child's age and individual assessment, which we were unable to analyze in the current study.

Interestingly, some participants changed their therapeutic services after transitioning to teletherapy. One reason for this could be that families may have found themselves reprioritizing their therapeutic needs given the changes forced upon them by the pandemic, resulting in IFSP changes after the transition to teletherapy. Some families had to balance work responsibilities with family obligations. This may have made it more challenging for families of children with multiple therapeutic needs to coordinate and attend to their child's therapy sessions. Furthermore, some households may only have one caregiver available, and others may have had multiple children that also needed the caregiver's attention, resulting in increasing demands of their time. A study by Murphy et al. (2021) suggested that parental dissatisfaction with teletherapy may be due to the increased burden of supporting their children as they adjusted to the changes required of telehealth. It is important to consider that some families may have their own preferences regarding their child's care (Camden, 2020). Families who chose to receive the same therapeutic services after transitioning to teletherapy may have done so as they had already worked with the same provider in person. The connection made between the family and provider during in-person sessions may have resulted in a greater comfort level with continuing EI services virtually. Through a hybrid in-person and virtual service delivery model, families and providers can benefit from increased convenience while maintaining the relationship benefits of an in-person interaction (Milne Wenderlich & Herendeen, 2021), creating a more successful EI experience. The desire of families to have their children partake in some form of therapy rather than nothing at all may have also resulted in IFSP changes with the introduction of new services. The increase in the number of children receiving speech therapy after transitioning to teletherapy could be due to the availability of more speech therapists in comparison to other providers. All the aforementioned factors may have potentially contributed to how caregivers made their decision regarding which EI services to continue during the pandemic.

Our findings have important implications with respect to the problem of provider shortage and ensuring that EI participants receive timely services (Fung & Ricci, 2020). Teletherapy can increase the number of available providers for families who reside in remote areas and minimize the amount of time from EIP intake to when families start receiving therapeutic services (Cason, 2009; Fung & Ricci, 2020). In fact, both families and providers have cited increased accessibility and flexibility as significant benefits of telehealth (Behl et al., 2017; Blaiser et al., 2013; Cole et al., 2019; Lalios, 2012; Olsen et al., 2012). Our study provides proof of efficacy in the provision of EI services via telehealth.

It is important to consider the relationship between caregiver and provider training and participant outcomes. In a study by Kronberg et al. (2021), goal achievement improved after a 9-week telehealth coaching intervention by trained EI providers. However, it is unclear whether the increase in child performance was associated with having a telehealth-trained provider, using a caregiver coaching model, or both. Previous studies have shown that a caregiver coaching approach may be effective in increasing child outcomes (Graham et al., 2013; Little et al., 2018). Additionally, several studies have found high caregiver engagement and self-efficacy with telehealth (Behl et al., 2017; Blaiser et al., 2013; Cole et al., 2019). A more recent study conducted during the COVID-19 pandemic found that there was both improved caregiver and provider perception of caregiver engagement and therefore confidence to implement treatment with telehealth (Philipps et al., 2021). The coaching model appears to enhance caregiver education, enabling caregivers to continue their child's therapy outside of formal EI sessions, which may then result in increased child outcomes.

### STRENGTHS AND LIMITATIONS

This study is one of the first to investigate the efficacy of telehealth in the setting of EI during the COVID-19 pandemic. In comparison to previous studies with a similar focus, this research study had a larger sample population and involved multiple therapy disciplines as well as various diagnosed conditions. This study also explored the efficacy of telehealth from two different perspectives: children who had both in-person therapy and teletherapy experience within the same therapy types, and children who utilized both service delivery models for different therapeutic services. However, all study participants received some type of EI service both in-person and virtually.

Despite the strengths of the current study, several limitations need to be considered. First, our sample population lacked socioeconomic diversity, minimizing the generalizability of the research findings. Our study population was predominantly Caucasian, spoke English as their primary language, and caregivers had higher educational levels. Second, this study measured participant progress using provider-reported data collected by New York State EIP. Standardized tools for measuring EI participant outcomes would provide more objective measurement of progress and increased validity to track progress uniformly. Third, this study did not account for provider differences in telehealth experience and delivery of virtual intervention. Although there may have been some providers who previously utilized teletherapy, there was no uniform training in the implementation of teletherapy among providers prior to its usage during the COVID-19 pandemic. Lastly, the study did not account for differences in EI enrollment duration among research participants prior to the adoption of teletherapy. Some participants may have been enrolled in EI longer than others, which may have resulted in a greater potential to achieve their goals virtually.

### FUTURE DIRECTIONS

Future studies investigating the efficacy of telehealth in EI require a larger sample population with increased socioeconomic diversity. It is important to explore the effect of race or ethnicity, socioeconomic status, diagnosed condition, and duration of EIP enrollment on EI participant outcomes. Future studies should also incorporate caregiver-reported measures in addition to provider-reported measures. Telehealth encourages caregivers to become more active participants by providing them a natural way to easily engage in their child's EI services. Lastly, it is important to determine for what therapy types and in what context telehealth could be successful.

## CONCLUSION

Telehealth may be a viable option for delivering EI services. Despite families and providers not having prior experience with telehealth, our research findings demonstrated that IFSP goals may be achieved virtually. Telehealth may provide a platform that is more efficacious for distinctive therapy types and may pose a challenge for others. More research is needed to identify which contextual factors make telehealth more effective. EI agencies should consider implementing a hybrid service delivery model, offering both in-person and telehealth services. Telehealth is a viable option in addressing provider shortage within the EI program and enhancing the overall EI experience for families and providers.
